# *Pneumocystis jirovecii* Pneumonia in Pediatric Inflammatory Bowel Disease: A Case Report and Literature Review

**DOI:** 10.3389/fped.2017.00161

**Published:** 2017-07-24

**Authors:** Sally J. Lawrence, Manish Sadarangani, Kevan Jacobson

**Affiliations:** ^1^Department of Pediatric Gastroenterology, Hepatology and Nutrition, BC Children’s Hospital, University of British Columbia, Vancouver, BC, Canada; ^2^Vaccine Evaluation Center, BC Children’s Hospital Research Institute, University of British Columbia, Vancouver, BC, Canada

**Keywords:** *Pneumocystis jirovecii*, pneumocystis pneumonia, inflammatory bowel disease, pediatric, opportunistic infection, immunosuppressive therapy, lymphopenia

## Abstract

Immunosuppressive therapy is a known risk factor for opportunistic infections. We report the first case of severe *Pneumocystis jirovecii* infection requiring intensive care in a pediatric patient with inflammatory bowel disease (IBD). The literature was reviewed and there were 92 reported cases of *Pneumocystis* pneumonia (PCP) in patients with IBD. Most sources were case reports and there was likely reporting bias toward patients receiving immunomodulators, anti-tumor necrosis factor (anti-TNF) therapy, and those who died. Overall, 56% of patients were males and 58% had Crohn’s disease. The median age was 45 years (interquartile range 30–68, range 8–78) and 86% of patients were lymphopenic. The case-fatality rate was 23%. Corticosteroids were used as IBD treatment in 88% of patients who subsequently developed PCP, 42% received thiopurines, 44% used anti-TNF therapy, and 15% received either cyclosporine or tacrolimus. Rates of mono, dual, triple, and quadruple immunosuppression therapy were 35, 35, 29, and 2%, respectively. This report highlights the importance of considering PCP in immunosuppressed lymphopenic pediatric IBD patients who present with unusual symptoms. Moreover, it should give gastroenterologists the impetus to limit immunosuppressive therapy to its minimal effective dose and consider options such as exclusive enteral nutrition wherever possible. Although there is no place for global PCP prophylaxis in IBD given the low incidence, in an era when there is increasing use of biologic agents with combination immunosuppressive therapy, the risk-benefit profile of PCP chemoprophylaxis should be revisited in selected cohorts such as patients on triple immunosuppression with corticosteroids, thiopurines, and a biological agent or calcineurin inhibitor, especially in lymphopenic individuals.

## Introduction

Immunosuppressive therapy is a known risk factor for opportunistic infections (1). We report a case that highlights the importance of considering opportunistic infection in immunosuppressed pediatric patients with inflammatory bowel disease (IBD) who present with unusual symptoms. We present the first case of severe *Pneumocystis jirovecii* (P. jirovecii) infection requiring intensive care in a pediatric patient with IBD.

## Case Report

A 12-year-old Caucasian girl was diagnosed with gastric and ileocolonic Crohn’s disease (CD), Paris classification L3, L4a, B1, G1, having presented with growth failure and abdominal pain and having undergone endoscopic and magnetic resonance enterography assessment. She failed exclusive enteral nutrition (EEN) and was treated with oral prednisone tapered over 3 months with maintenance azathioprine (AZA) (2 mg/kg/day). Treatment resulted in resolution of clinical symptoms and improved biochemical markers. She had recurrence of symptoms after 9 months with abdominal pain and diarrhea. Infective work-up was negative and she was commenced on oral budesonide 9 mg and her AZA dose was increased (2.5 mg/kg/day). Thiopurine metabolites at this stage were normal [6-TGN 278 pmol/8 × 10^8^ (normal range 230–400), 6-MMPN 734 pmol/8 × 10^8^ (normal range <5,700)].

**Figure 1:**
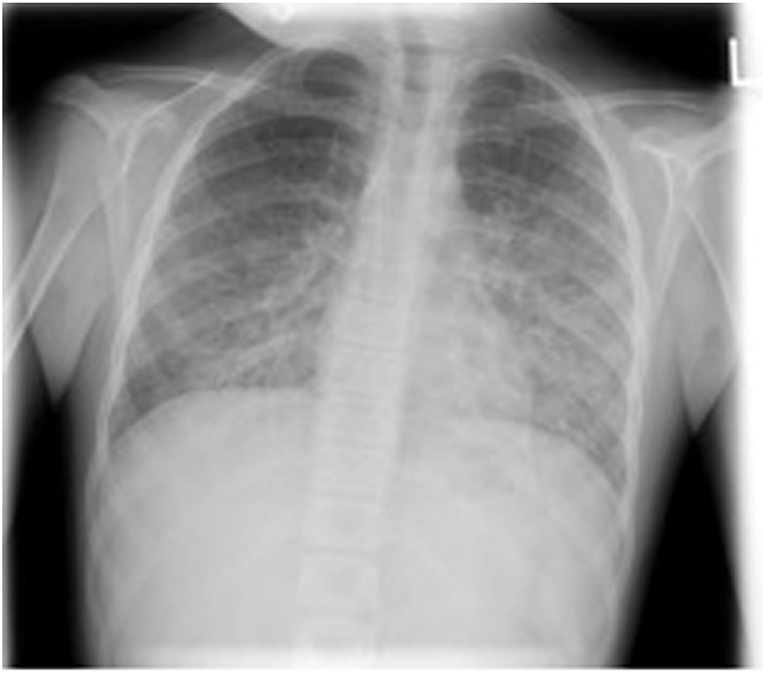
Chest X-ray demonstrating bilateral pulmonary infiltrates caused by pneumocystis pneumonia.

**Figure 2:**
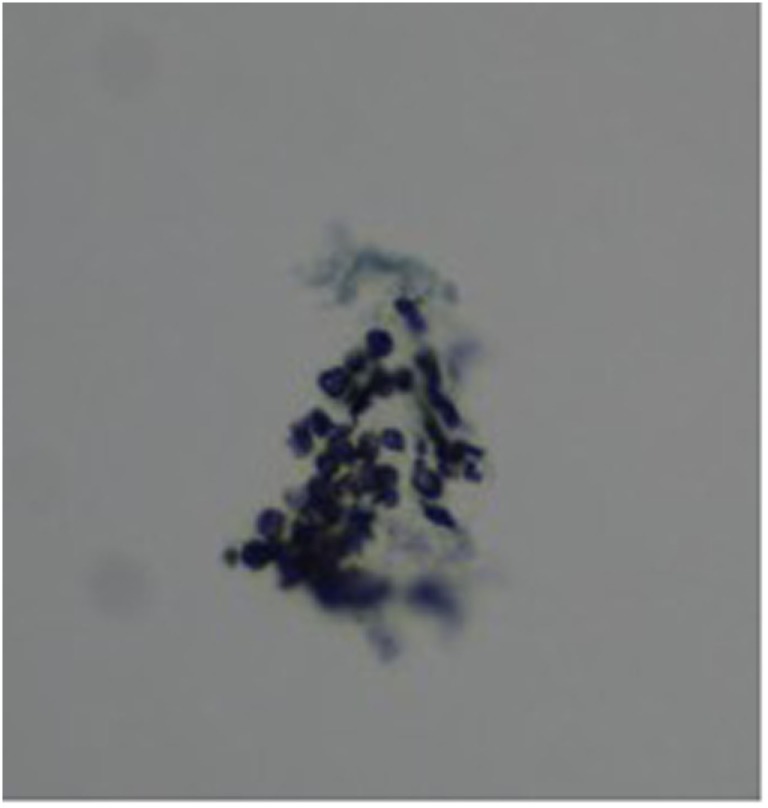
Grocott–Gomori’s methenamine silver stain of sputum specimen showing “cup shaped” *Pneumocystis jirovecii* cysts in small aggregates.

Three months later, she presented with a 3-day history of fever, dry cough, and progressive dyspnea. On examination, she was hypoxic with SaO_2_ 90% despite 15 l/min high flow oxygen. She had severe lymphopenia (0.0 × 10^9^/l on manual count), elevated white cell count (10.8 × 10^9^/l), raised C-reactive protein (268 mg/l), and lactate dehydrogenase was 763 U/l. Chest X-ray showed bilateral interstitial infiltrates (Figure 1). She required intensive care for bi-level positive airway pressure respiratory support. AZA and budesonide were discontinued and she was commenced on piperacillin–tazobactam, clarithromycin, and oseltamivir to provide empiric coverage against bacteria (including atypical) and influenza, with minimal improvement in symptoms. An induced sputum sample was negative for bacterial and viral pathogens [culture and broad panel polymerase chain reaction (PCR)] but revealed *P. jirovecii* on silver stain (Figure 2). Intravenous (IV) trimethoprim–sulfamethoxazole (TMP–SMX) 20 mg/kg TMP/100 mg/kg SMX and methylprednisone 1 mg/kg twice daily were initiated, which resulted in improvement in respiratory status and weaning of respiratory support over 7 days.

Prior to discharge, she was investigated for an underlying primary immunodeficiency. Human immunodeficiency (HIV) testing was negative and immunoglobulin levels were normal. B and T cell panel revealed a low absolute count of CD3, CD4, and CD8 (0.47, 0.36, 0.14 × 10^9^/l respectively) with normal number of B cells (0.43 × 10^9^/l), normal CD4/CD8 ratio (2.43), and a good response to previous diphtheria and tetanus vaccines. She remained lymphopenic during admission, but this slowly improved. Notably, at IBD diagnosis, she had normal lymphocyte levels [lymphocyte count 1.7–2.6 (normal range 0.9–3.5 × 10^9^/l)]; however, after AZA initiation, she had intermittent lymphopenia (0.4–1.2 × 10^9^/l). Thiopurine methyltransferase activity testing was unfortunately unavailable at diagnosis.

She was discharged on oral TMP–SMX for 21 days, having had 3 days of IV therapy. She received 3 days of methylprednisone followed by 5 days of oral prednisone, which was tapered over 2.5 months. AZA was restarted at a reduced dose (1.5 mg/kg/day). Within 2 months, she had normal lymphocyte numbers with a normal absolute CD4 count (0.56 × 10^9^/l) and normal T-cell numbers. She developed an urticarial rash thought to be secondary to TMP–SMX 4 days post discharge and was changed to oral clindamycin and primaquine to complete the 21-day treatment course. She was then started on prophylactic oral dapsone, which was discontinued when she developed arthralgia. She has not subsequently taken PCP prophylaxis.

One year post-PCP admission, she was commenced on adalimumab monotherapy due to ongoing poor growth, abdominal pain, and an elevated fecal calprotectin (>1,800 μg/g). This resulted in resolution of symptoms, catch-up growth, and progression through puberty. She has had no further significant infections in the last 5 years and her lymphocyte count has remained stable.

## Literature Review and Discussion

### Overview of *P. jirovecii* Pneumonia

*Pneumocystis jirovecii* (formerly known as *Pneumocystis carinii*) is a ubiquitous opportunistic fungus, which causes pneumonia [*Pneumocystis* pneumonia (PCP)]. It was first described in 1940s malnourished infants (2). In the 1980s, it was associated with HIV-infected patients with low CD4 counts (3). PCP continues to be an acquired immune deficiency syndrome-defining illness although the advent of antiretroviral therapy has resulted in incidence reduction (4). PCP has been recognized as a disease in children with primary cell-mediated immunodeficiency, patients receiving chemotherapy for hematological malignancies, solid organ, and bone marrow transplant recipients and in patients requiring immunosuppressants (5). Notably, the incidence in the immunosuppressed non-HIV population is increasing with the escalating use of immunosuppressive agents (4, 6, 7). Inflammatory autoimmune conditions such as IBD account for up to 20% of PCP in HIV-negative patients (8).

*Pneumocystis jirovecii* exposure appears to occur early in life and is often asymptomatic (9, 10). Reinfection of the immunosuppressed host through environmental or person-to-person transmission rather than reactivation from latency appears to be the major mode of acquisition in immunosuppressed patients (10). Effective host defenses against *P. jirovecii* are mediated by the innate, T-cell, and to a lesser extent, humoral immune responses. In immunosuppressed patients, the infection results in a dysfunctional immune response, composed of mononuclear cells, CD8 lymphocytes, and activated macrophages, which causes diffuse lung damage (11). Moreover, the inhaled *Pneumocystis* trophozoites inhibit epithelial repair processes within the alveoli resulting in severe lung damage (12).

Adult data show that unlike in HIV-infected individuals where presentation can be slow and insidious, non-HIV infected immunocompromised individuals can have an aggressive course often culminating in respiratory failure over several days (4, 13–15). PCP is characterized by a non-productive cough, fever, and dyspnea. On chest X-ray, diffuse bilateral interstitial pulmonary infiltrates are most common (4). In equivocal cases, high resolution chest computed tomography is more sensitive and commonly shows diffuse ground glass opacity (16).

*Pneumocystis jirovecii* cannot be cultured, therefore, definitive diagnosis is made by visualization of the organism’s cysts and trophozoites. This can be achieved using induced sputum, bronchioalveolar lavage specimens, or lung tissue stained with Grocott–Gomori’s methenamine silver or visualized using immunofluorescence. Unfortunately, sensitivity is <67% in the non-HIV infected patient due to low numbers of *P. jirovecii* (17). PCR testing of *P. jirovecii* nucleic acid in respiratory samples is more sensitive for diagnosis; however, discrimination between colonization and actual disease has been challenging. Quantitative PCR is thought to help differentiate between these two entities better than qualitative PCR with recent research focusing on oral washes as a less invasive diagnostic tool (18, 19).

First line treatment of PCP is IV TMP–SMX (15–20 mg/kg/24 h of the trimethoprim component in three divided doses) due to excellent tissue penetration, rapid response, and low cost (4). Adverse reactions include leukopenia, thrombocytopenia, and rashes including Stevens–Johnson syndrome (5). However, reactions appear less common in children (20). Alternative therapy includes clindamycin plus primaquine, pentamidine, and dapsone (21). Atovaquone has been used for mild disease as it is less effective than TMP–SMX, but better tolerated (22). Corticosteroids have been used as an adjuvant treatment in severe disease, reducing respiratory failure and the length of intensive care unit stay (23).

PCP mortality rates are between 20 and 60% in immunocompromised non-HIV infected individuals in contrast to rates of 10–20% in HIV-infected patients (4, 7, 13, 15, 24, 25). The more severe course is thought to be secondary to a more disseminated pulmonary inflammatory response and diagnostic delay (4, 26).

### PCP and IBD

A retrospective cohort study determined that IBD patients were at an elevated risk of PCP compared to the general population with an increased relative risk (hazard ratio 2.96; 95% CI 1.75–4.29) but low absolute risk (0.03%) (26). The incidence of PCP in immunosuppressed IBD patients [thiopurines, methotrexate, calcineurin inhibitors, anti-tumor necrosis factor (anti-TNF) agents, or steroids] was 32/100,000 patient-years (PY) compared to 5.5/100,000 PY for non-immunosuppressed IBD patients (26). The risk appeared greater in CD compared with ulcerative colitis (UC) (26). In another population-based IBD cohort, double immunosuppression resulted in a higher risk of PCP than monotherapy (0.6/100 vs. 0.3/100 PY). There were few patients on triple therapy making risk analysis challenging (27).

There is only one published case of a child with IBD developing PCP. The 8-year-old CD patient, on infliximab monotherapy, developed PCP with concurrent disseminated histoplasmosis after 15 months therapy. His disease course was not severe. He did not require respiratory support and responded to TMP–SMX. He was maintained on budesonide with no PCP prophylaxis (28). PCP has been described in adult IBD patients on corticosteroids, calcineurin inhibitors, thiopurines, and anti-TNF agents (8, 26, 29–33).

We performed a literature review of PCP in IBD. A database search for studies on MEDLINE, EMBASE, and the Cochrane Controlled Trials Registry was performed. References of included articles were searched for further studies. Table 1 summarizes the details of published IBD studies reporting patients with PCP. Most sources were case reports [evidence level 5 (34)], and there was likely reporting bias toward immunomodulators, anti-TNF therapy, and patients who died. There were 92 reported cases of PCP in patients with IBD in the English literature, of which 56% were males and 58% had CD. The median age was 45 years [interquartile range (IQR) 30–68, range 8–78]. There was little documented information about CD4 counts at PCP diagnosis; however, 86% (12/14) of patients were lymphopenic. The case-fatality rate (CFR) was 23% (7/31) based on the reported outcome data. Where medication was documented, rates of mono, dual, triple, and quadruple immunosuppression therapy were 35% (18/52), 35% (18/52), 29% (15/52), and 2% (1/52), respectively. The numbers were too small to comment on the effect of incremental risk with increasing numbers of immunosuppressants. Corticosteroids, as mono, dual or triple therapy, were used as IBD treatment, in 88% (46/52) of patients who developed PCP. 42% (22/52) received thiopurines, 44% (23/52) took anti-TNF therapy, and 15% (8/52) used either cyclosporine or tacrolimus.

**Table 1:** Summary of published literature of inflammatory bowel disease patients who developed pneumocystis pneumonia (*n* = 92).

Reference	No of patients	Disease subtype	Gender	Age (years)	Medication at time of PJP	Single (S), dual (D), triple (TR), quadruple (Q) immunosuppression	Outcome
Khatchatourian and Seaton (30)	1	UC	M	68	CS + T	D	Died
Lee et al. (35)	1	UC	M	21	CS + T	D	Survived
Takenaka et al. (36)	3	UC (100%)	F (67%)	26–68	CS + T (67%), CS (33%)	S (33%)	Survived (100%)
						D (67%)	
Bernstein et al. (29)	2	UC (100%)	M (100%)	32–73	CS (100%)	S (100%)	Died (50%)
Escher et al. (32)	2	UC (100%)	M (100%)	72–74	CS + Tac, CS + Tac + T	D (50%)	Died (100%)
						TR (50%)	
Art et al. (37)^a^	3	UC (100%)	M	32	CS + CSA + T (100%)	TR (100%)	Died (33%)
Quan et al. (38)	1	UC	M	63	CS + CSA	D	Died
Scott et al. (31)	1	UC	M	43	CS + CSA	D	Survived
Smith and Hanauer (39)	1	UC	M	32	CS + CSA	D	Survived
Desales et al. (8)	1	CD	M	36	CS + T + anti-TNF	TR	Survived
Lawrance et al. (40)	2	CD (100%)	F (50%)	18–32	CS + T + anti-TNF, CS + MTX + MMF + anti-TNF	TR (50%)	Survived (100%)
						Q (50%)	
Cotter et al. (27)	3	UC (67%)	M (100%)	63–78	MTX + anti-TNF, CS + anti-TNF, T	S (33%)	Survived (100%)
						D (67%)	
Tschudy and Michail (28)	1	CD	M	8	Anti-TNF	S	Survived
Iwama et al. (41)	1	CD	M	51	Anti-TNF	S	Survived
Velayos and Sandborn (42)	1	CD	M	19	T + anti-TNF	D	Survived
Kaur and Mahl (43)	1	CD	M	59	CS + anti-TNF	D	Died
Stratakos et al. (44)	1	CD	F	77	CS + anti-TNF	D	Survived
Estrada et al. (45)	1	UC	M	45	CS + T + anti-TNF	TR	Survived
Sharma and Rao (46)	1	CD	F	36	CS + T + anti-TNF	TR	Survived
Seddik et al. (47)	1	CD	M	29	CS + T + anti-TNF	TR	Survived
Itaba et al. (48)	1	CD	F	57	CS + T + anti-TNF	TR	Survived
DeFilippis et al. (49)	1	CD	F	56	CS + MTX + anti-TNF	TR	Survived
Long et al. (26)	38	CD (55%) UC (40%)	F (55%)	43–57 IQR	CS: 11/38	S: 12/38 (32%)	ND
					T: 1/38	D: 5/38 (13.2%)	
					T + CS: 5/38	TR: 4/38 (10.5%)	
					CS + 2IM: 2/38		
		ND (5%)			CS + IM + anti-TNF: 2/38		
Kaur and Mahl (33)	16	CD (88%)	ND	ND	Anti-TNF (100%)	ND	ND
Fillatre et al. (14)	1	ND	ND	ND	ND	ND	ND
Bienvenu et al. (7)	4	ND	ND	ND	ND	ND	ND
Roblot et al. (50)	2	ND	ND	ND	ND	ND	ND

*CS, corticosteroids; T, thiopurines (azathioprine/6-Mercaptopurine); Tac, tacrolimus; CSA, cyclosporine; anti-TNF, anti-tumor necrosis factor therapy (adalimumab or infliximab); MTX, methotrexate; MMF, mycophenolate mofetil; IMs, immunomodulators (thiopurine, tacrolimus); ND, not documented; CD, Crohn’s disease; UC, ulcerative colitis; IQR, interquartile range*.

*^a^Data on gender and age only available for the one patient who died*.

### PCP and Corticosteroids

Corticosteroids are known to reduce CD4 lymphocytes, which predisposes to PCP development (51). Corticosteroids have emerged as a major contributor to PCP in the non-HIV immunosuppressed population and the risk is particularly increased at or above 16 mg of prednisolone (50–52). The median therapy duration prior to PCP was 8–12 weeks (50, 51). Interestingly, in some studies, the disease only became apparent when corticosteroids were tapered (24, 50, 51).

### PCP and Thiopurines

Thiopurines (AZA and 6-mercaptopurine) can inhibit cell-mediated immunity, which influences PCP development. In the literature, two IBD patients on thiopurine monotherapy developed PCP, 10 were on dual therapy, as was the case with our patient, and 10 were on triple therapy. The CFR in this group was 19% (3/16), where outcome data were available.

### PCP and Calcineurin Inhibitors

Cyclosporine works by inhibiting production of IL-2 by helper T-cells and by affecting T-cell, B-cell, neutrophil, and mast cell function. In the literature, 50% (4/8) of IBD patients with PCP died (32, 37, 38). Such cases have prompted a discussion regarding the role of prophylactic antibiotics in patients on cyclosporine; however, the limited cases and reporting bias needs to be considered (37, 53, 54).

### PCP and Methotrexate

Three patients in the IBD literature developed PCP while on methotrexate in combination with corticosteroid and anti-TNF therapy. Methotrexate has been implicated in the development of PCP in rheumatoid arthritis with 28 documented cases, 25% of whom died (55, 56).

### PCP and Anti-TNF Agents

Cytokines inhibited by anti-TNF agents are involved in the host response to PCP resulting in reduced PCP clearance. Moreover, anti-TNF therapy can lower CD4 counts making patients more susceptible to PCP (57). Review of the Food and Drug Administration Adverse Event Reporting System data between 1998 and 2003 identified 84 patients with PCP associated with infliximab, with a mean age of 55 years; 19% of patients had IBD. Concomitant immunosuppressive agents included immunomodulators (66%), corticosteroids (50%), and cyclosporine (5%). The CFR was 27% (33). PCP has been reported to occur 9–14 weeks after the first infliximab induction dose (33, 57, 58). Notably, Colombel et al. did not report any cases of PCP associated with 3,160 patients (in six global clinical trials) on adalimumab (59). However, there are two case reports of PCP in IBD patients on adalimumab (8, 40). In total, 162 cases of PCP are reported in the IBD and rheumatology literature associated with anti-TNF therapy, which includes pediatric patients. Of the 138 patients with outcome information available, 20% died. Unfortunately, studies do not always specify whether single, double, or triple immunosuppression was used (8, 26–28, 33, 40–49, 56, 58, 60, 61). Data on the incidence of PCP in patients on anti-TNF agents are largely based on rheumatoid arthritis studies and most are population based. The majority of studies report incidence rates <50 cases per 100,000 PY (62). Results are hampered by heterogeneity in the method of PCP diagnosis, moreover, discrimination between *P. jirovecii* colonization and actual disease can be challenging.

There are no published data on PCP associated with vedolizumab, ustekinumab, or other novel IBD treatments (63, 64). More long-term data are required to assess safety profiles of these agents.

### Other Risk Factors for PCP in Non-HIV Infected Immunosuppressed Patients

The use of multiple immunosuppressive agents incrementally increases the risk of opportunistic infection in IBD; moreover, malnutrition and surgery can play a role, although this has not been specifically addressed in PCP (1, 27, 40, 51, 52, 62). As documented in our case, lymphopenia (especially CD4 count <300 cells/mm^3^) has been associated with increased risk of PCP in 60–95% of cases (5, 24, 50, 65). Notably, not all immunosuppressed patients will present with lymphopenia (24, 65).

Long et al. reviewed a case series of 38 IBD patients who developed PCP and showed that rates of hospitalization at some stage within 60 days of PCP were high (50%) (26). Moreover, patients had higher rates of comorbidities, principally, lung disease and diabetes mellitus, compared to the background population. Advanced age >65 years was found to be an additional risk (26).

### PCP in Other Pediatric Non-HIV Immunosuppressive Conditions

A population-based cohort study in juvenile idiopathic arthritis patients reported an incidence of 7/100,000 PY (66). Multiple immunosuppressive agents and lymphopenia are important risk factors in rheumatic diseases (67). The risk of PCP in pediatric cancer depends on the malignancy type and chemotherapy category. Lymphoid malignancies have the highest risk with rates of 22–45% (68). The overall risk of PCP post solid organ transplant has been estimated to be 5–15% in the absence of PCP prophylaxis (5). Risk factors include malnutrition, previous cytomegalovirus infection, and underlying lung disease. Medications such as steroids, antilymphocyte agents, calcineurin inhibitors, and biological agents such as alemtuzumab (anti-CD52 monoclonal antibody) have also been implicated (5).

### Prophylaxis against PCP

In a 2012 survey of PCP prophylaxis practice by gastroenterologists, 11% prescribed prophylaxis to their patients with IBD on combination therapy. Gastroenterologists were more likely to prescribe if they had previous practical experience of PCP, or practiced in an academic center (69).

The effects of PCP can be severe, however, prevention entails using drugs with adverse effects that may counterbalance the benefits, as occurred in our patient. The guidelines for PCP prophylaxis in HIV-infected patients have been universally adopted, but there is a lack of consensus on prophylaxis with TMP–SMX in IBD patients. A recent Cochrane meta-analysis of prophylactic treatment with TMP–SMX in non-HIV infected patients included 1,412 patients, of which 520 were children. The authors were unable to find published literature addressing prophylaxis in IBD. In patients with hematological cancers and transplant recipients, they reported an 85% reduction in PCP incidence with prophylaxis and PCP-related mortality reduced by 83% with few adverse events reported (20). No children in the included studies had a severe adverse event compared to 3.1% of adults suggesting a lower probability of harm in children.

There is insufficient evidence to recommend PCP prophylaxis for all IBD patients on immunosuppressive monotherapy; however, with increasing number of immunosuppressive agents, the risk of opportunistic infection is known to increase (1, 27, 62). Large studies addressing the specific incremental increased risk associated with multiple immunosuppressive agents especially triple therapy have not been done. This needs to be considered, as the use of multiple immunosuppressive agents are increasing. A recent study showed that approximately half of patients started on anti-TNF therapy were already on two immunosuppressive agents (40). Dietary therapies (EEN, partial enteral nutrition, and exclusion diets) in CD are increasingly being used and investigated and are attractive options to limit immunosuppression and improve nutrition in this population (70–72).

A study using simulation modeling to address cost and comparative effectiveness of PCP prophylaxis in CD concluded that at the present incidence, routine chemoprophylaxis was not cost effective but, based on limited data, it may be effective in triple immunosuppressive therapy (73). The 2014 European Crohn’s and Colitis opportunistic infection guidelines recommend PCP prophylaxis in IBD patients on triple immunosuppression including either a calcineurin inhibitor or anti-TNF therapy. This recommendation is based on expert gastroenterology and infectious disease opinion (74). Other practical approaches proposed for patients on high-dose steroids and multiple immunosuppressants include measurement of CD4 counts in those patients with a total lymphocyte count of <600 cells/mm^3^ as a means of identifying patients at risk of infections, although not all patients who develop PCP have lymphopenia, therefore, precluding its use as an isolated risk identification guide (24, 43, 65, 75). Evaluating the utility of measuring CD4 counts in IBD patients on multiple immunosuppressants should be considered.

## Conclusion

We described a case of severe PCP in an immunosuppressed lymphopenic pediatric IBD patient. This case reiterates the importance of limiting immunosuppression to its minimal effective dose. Although the incidence of PCP in the IBD population is low, it is an aggressive condition that has a higher relative risk in IBD patients compared to the general population. Moreover, it is associated with significant morbidity and mortality. PCP should be considered in the differential diagnosis of immunosuppressed pediatric IBD patients who develop respiratory symptoms, with a low threshold for treatment. Although there is no place for global PCP prophylaxis in IBD, in an era when there is increasing use of biologic agents with combination immunosuppressive therapy, the risk-benefit profile of PCP prophylaxis should be revisited in selected cohorts such as patients on triple immunosuppression with corticosteroids, thiopurines, and a biological agent or calcineurin inhibitor especially in lymphopenic individuals. Further studies are required to guide definitive PCP prophylaxis in high risk subgroups of IBD patients.

## Informed Consent

Written informed consent for publication of the case report and figures was obtained from the patient and parents.

## Author Contributions

SL performed the literature review and wrote the manuscript. MS and KJ critically reviewed the manuscript.

## Conflict of Interest Statement

The authors declare that the research was conducted in the absence of any commercial or financial relationships that could be construed as a potential conflict of interest.
